# Prevalence and relationship with health of off-label and contraindicated drug use in the United States: a cross-sectional study

**DOI:** 10.1080/20523211.2025.2472221

**Published:** 2025-03-06

**Authors:** Katharina E. Blankart, Frank R. Lichtenberg

**Affiliations:** a Faculty of Economics and Business Administration, University of Duisburg-Essen, Essen, Germany; b Business School, Columbia University in the City of New York, New York, NY, USA; c National Bureau of Economic Research, Cambridge, MA, USA

**Keywords:** Prescription drug use, off-label drug use, contraindications, health care utilisation, DrugCentral database, Medical Expenditure Panel Survey, regulatory pathways, behavioural economics

## Abstract

Background: Off-label and contraindicated prescription drug use can result in adverse health outcomes. Despite concerns, the extent and characteristics of such usage patterns remain underexplored in the American population. We conducted a cross-sectional study analysing outpatient prescription data between 2016 and 2021 to determine the prevalence of off-label and contraindicated drug use.

Methods: The study used labelling information from DrugCentral and the Medical Expenditure Panel Survey, focusing on the American non-institutionalised population. We analysed 9872 drug-indication and 34,138 drug-contraindication pairs among 46,770 patients and 1,596,753 prescriptions. Linear and probit regressions, and a double machine learning approach, were employed to assess associations between off-label/contraindicated use, health status, and healthcare utilisation, adjusting for demographic and health-related factors.

Results: Overall, 75% of prescriptions were for labelled indications, while 25% were off-label; 54% were contraindicated. Only 33% of prescriptions were both indicated and not contraindicated. Off-label prescriptions had a lower contraindication rate (48.8%) compared to indicated prescriptions (56.2%). Improved health status and reduced medical expenditure correlated with lower off-label prescription rates. Notably, newer drugs (post-1997) had a higher rate of prescriptions that were both indicated and not contraindicated (43%) compared to older drugs (pre-1979, 21%). Patterns of off-label and contraindicated use were consistent across racial and educational demographics.

Conclusion: Off-label and contraindicated drug use is prevalent in outpatient prescriptions and is associated with worse health outcomes and increased healthcare utilisation. These findings suggest a need for enhanced monitoring and regulatory measures to minimise risks associated with inappropriate prescription practices.

## 1. Background

When the U.S. Food and Drug Administration (FDA) approves a new drug, it specifies the drug’s appropriate use. The drug’s indications are defined in the approved label, which is the official description of a drug product and includes the indications for which the drug should be used (Food and Drug Administration, 2023). The FDA also specifies when a drug is contraindicated, which refers to clinical situations for which the risk from use outweighs any possible therapeutic benefit (U.S. Department of Health and Human Services et al., 2011). When drugs are used outside these definitions, they may often be used inappropriately: patients may not have the conditions for which the drugs they use are indicated, and they may have conditions for which the drugs they use are contraindicated.

Some discrepancies between the way drugs are actually used and their appropriate uses may be due to the behaviour of physicians, who are free to prescribe drugs off-label, i.e. for conditions not included in the drug’s label. Seetasith et al. (2017) showed that policy and labelling interventions to reduce inappropriate prescribing of erythropoiesis-stimulating agents did not reduce prescribing of unsupported indications. Other discrepancies may be due to the behaviour of patients. Rice (2013) argued that people often make decisions in health care that are not in their best interest, ranging from failing to enrol in health insurance to which they are entitled, to engaging in extremely harmful behaviours. Behavioural economics, a mixed discipline covering economics, psychology, and neuroscience, suggests that individuals frequently deviate from rational behaviour (Matjasko et al., 2016).

Using the wrong combination of prescription drugs or the wrong technology can have negative health and economic consequences for patients (Glied & Sacarny, 2018). Non-optimised prescription drug use caused by treatment failures or new medical problems due to prescription drug treatments have been estimated to cost $528.4 billion annually (Watanabe et al., 2018).

Several studies have examined the prevalence and characteristics of off-label drug use across a large number of diseases and based on registry or physician prescription data. 21% of U.S. prescriptions for 160 commonly prescribed drugs were off-label (Radley et al., 2006). Based on 2005–2009 data from Quebec, 11% of prescriptions were off-label (Eguale et al., 2016). Based on data from the National Ambulatory Medical Care Survey, the rate of off-label use of prescription drugs in the U.S. increased from 29.9% in 1993 to 38.3% in 2008 (Bradford et al., 2018). 41% of cancer drug utilisation was off-label based on MarketScan data from 1997–2007, of which 17% were defined inappropriate whereas 29% were considered appropriate uses (Smieliauskas et al., 2018).

Off-label drug use may only be desirable if it benefits patients. Radley et al. (2006) argued that ‘most off-label drug mentions … had little or no scientific support,’ and that ‘efforts should be made to scrutinize under-evaluated off-label prescribing that compromises patient safety or represents wasteful medication use.’ That study identified drug, patient, and physician characteristics to be associated with off-label use which may be undesirable. Bradford et al. (2018) found ‘off-label prescribing patterns by physicians that were consistent with enhancement of patient welfare.’ They found that Medicaid patients are more likely to be prescribed off-label. In a study of cancer patients in Switzerland, 57% of requests to use cancer treatments were off-label, there was no supporting evidence for benefit, and the probability of receiving reimbursement was not higher if there was supporting evidence from clinical trials (Herbrand et al., 2021). Nevertheless, there may be off-label uses that are equal or superior to the comparable on-label options (Katsanos et al., 2022).

Contraindicated drug use is identified from clinical trials or post-approval market surveillance data. In the UK, computerised medication records indicate that contraindicated drug use is relatively rare, occurring in 1.9 per 1000 patient-years or 4.3 per 1000 patients prescribed multiple drugs annually (Chen et al., 2005). However, Americans typically use more than one doctor. Data from patients with chronic heart failure show that 23.8% received at least one inappropriate drug (Maggioni et al., 2007). Measuring the prevalence of contraindicated drug use could reveal how incorrect drug combinations contribute to mortality and morbidity.

While previous studies have examined either off-label use or contraindicated prescribing considering drug–drug interactions in isolation, the prevalence of these two distinct types of inappropriate medication use linked to certain diseases remains poorly understood. Most research on contraindicated drug use is on drug–drug interactions (Eljaaly et al., 2019) and has considered adverse drug reactions as the only measure of health outcomes (Eguale et al., 2016). This knowledge gap is particularly concerning for patient safety and health care use, as both off-label and contraindicated prescribing represent different aspects of non-optimal drug use that could independently contribute to adverse health outcomes that are related to inefficiencies in health care. A large-scale, population-based study using comprehensive prescription data is needed to systematically examine patterns of medication use and their associations with health care outcomes which could inform clinical practice and regulatory policy. We performed a cross-sectional study of 46,770 patients using outpatient prescription medication to measure the prevalence and characteristics of off-label and contraindicated use, relying on publicly available Medical Expenditure Panel Survey (MEPS) data on a large, representative sample of Americans.

## 2. Methods

### 2.1. Study design and setting

This study examines the extent of off-label and contraindicated outpatient prescription drug use in the US non-institutionalised population and its associations with health status and healthcare utilisation. We use 2016–2021 Medical Expenditure Panel Survey data and DrugCentral data, and analyse 9872 drug-indication and 34,138 drug-contraindication pairs among 46,770 patients with 1.6 million prescriptions.

We performed a cross-sectional study of 348 drugs by 474 condition combinations captured in the DrugCentral database that reflect drugs approved by the Food and Drug Administration that individuals included in the MEPS reported to have been prescribed. We studied prescriptions reported in the MEPS between 2016 and 2021 because after 2015, the MEPS allowed us to readily link 348 drug codes.

### 2.2. Sources and tools

We construct data that enable us to examine the prevalence of off-label and contraindicated use. The data allow us to study associations between off-label use, some patient characteristics including race, educational attainment, insurance coverage, health status and utilisation. In addition to determining whether each of 1.6 million prescriptions was off-label, we can determine whether each prescription was *contraindicated* for the patient, based on his or her medical conditions.

Data on drug indications and contraindications were constructed from the DrugCentral database (Avram et al., 2021, 2023; Ursu et al., 2017, 2019), an online drug information resource created and maintained by the Division of Translational Informatics at the University of New Mexico in collaboration with the Illuminating the Druggable Genome Consortium, a U54 programme funded by the NIH Common Fund. DrugCentral initially extracted data on 10,707 indications and 27,851 contraindications from the Observational Medical Outcomes Partnership Common data model version 4.4. Since this project transitioned to Observational Health Data Sciences and Informatics (2024), updated drug indication and contraindication data are covered under a revised license agreement that requires subscription licenses. Indications for drugs approved after 2012 (322 pairs) were extracted from approved drug labels and mapped onto SNOMED-CT and UMLS concepts. We used SNOMED CT to ICD-10-CM Mapping Resources to map SNOMED CT to ICD-10-CM (National Library of Medicine, 2024).

Using the DrugCentral and SNOMED data, we constructed data on the indications of 2375 drugs for 795 (3-digit ICD10) diseases, and on the contraindications of 1412 drugs for 684 diseases. The data we constructed appear to be generally consistent with lists of drugs approved for different types of cancer published by the National Cancer Institute (2024). As a result, we obtained 9872 unique drug-indication and 34,138 drug-contraindication pairs. For example, for omeprazole, which is the most commonly prescribed drug (70,388 prescribed medicine events in our sample), there are nine indications and 17 contraindications.

Data on prescription drugs used by, medical conditions borne by, and other characteristics of patients were obtained from the MEPS, a set of large-scale surveys of families and individuals, their medical providers, and employers across the United States (Cohen et al., 2009). We use data from the Household Component, which collects data from a sample of families and individuals in selected communities across the United States, drawn from a nationally representative subsample of households. During the household interviews, MEPS collects information for each person in the household on the following: demographic characteristics, health conditions, health status, use of medical services, charges and source of payments, access to care, satisfaction with care, health insurance coverage, income, and employment.

We use data from four different MEPS files in each of 6 years (2016–2021; 24 files total): Prescribed Medicines Files, Medical Conditions Files, Appendices to MEPS Event Files (CLNK files), and Full Year Consolidated Data Files.

*MEPS Prescribed Medicines Files* provide detailed information on household-reported prescribed medicines for a nationally representative sample of the civilian noninstitutionalised population of the United States. Each record in the event files represents a unique prescribed medicine event; that is, a prescribed medicine reported as being purchased or otherwise obtained by the household respondent, and includes the following: an identifier for each unique prescribed medicine; detailed characteristics associated with the event (e.g. national drug code (NDC), medicine name, selected Multum Lexicon variables, etc.); the date on which the person first used the medicine; total expenditure and sources of payments; and types of pharmacies that filled the household's prescriptions.

*MEPS Medical Conditions Files* provide information on household-reported medical conditions. Medical conditions reported by the Household Component respondent were recorded by the interviewer using a condition pick-list with ICD-10-CM codes already assigned to conditions in the list. Reported conditions not in the pick-list were recorded as verbatim text and then were coded to ICD-10-CM codes (ICD10CDX) by professional coders. Each record represents one *current* medical condition reported for a household survey member who resides in an eligible responding household and who has a positive person or family weight. A condition is defined as *current* if it is linked to an event during the survey year.

*Appendices to MEPS Event Files (CLNK files)* are used for linking the MEPS Conditions Files with the MEPS Prescribed Medicines Files.

*MEPS Full Year Consolidated Data Files* contain data on demographics, access to care, patient satisfaction, health status, disability days, quality of care, employment, and health insurance.

Over 1/3 of the 77,234 individuals in the MEPS 2016–2021 had zero prescriptions. We analyse the prescriptions of the 46,770 individuals who had one or more prescriptions.

### 2.3. Definitions of drug use categories

We examine two types of potentially inappropriate drug use: prescriptions indicated for medical conditions that the patient does not have (off-label use) and prescriptions contraindicated for medical conditions that the patient does have (contraindicated use). These represent distinct types of potentially inappropriate medication use – the former lacking evidence of benefit, the latter having evidence of risk.

When the FDA approves a drug, it specifies two key aspects of appropriate use: indications and contraindications (U.S. Department of Health and Human Services et al., 2011; US Food and Drug Administration, 2019). Indications define the medical conditions for which clinical trials have demonstrated the drug's safety and efficacy, and these are listed on the FDA-approved label. Off-label use occurs when a drug is prescribed for conditions not included in these approved indications. While off-label prescribing is legal and common in medical practice, it means the drug is being used without the same level of evidence required for FDA-approved indications (Richardson, 2016).

In contrast, contraindications identify specific clinical situations where the drug has been shown to pose risks that outweigh potential benefits. These contraindications, also specified on the FDA label (U.S. Department of Health and Human Services et al., 2011), are based on evidence of harm from clinical trials or post-market surveillance. While off-label use represents prescribing without explicit evidence of benefit, contraindicated use represents prescribing despite evidence of potential harm. The National Cancer Institute (2011) defines a contraindication as any reason (such as a symptom or medical condition) that makes a treatment potentially harmful. This study focuses on drug-disease contraindications, where a patient's existing medical condition makes certain medications potentially harmful. Drug-disease contraindications are particularly important for patient safety as they represent situations where a drug may worsen an existing condition, interfere with its treatment, or pose heightened risks for patients with specific diseases. For example, certain antipsychotic medications are contraindicated in patients with dementia due to increased mortality risk and worsening of cognitive functions (‘FDA,’ 2008).

### 2.4. Measures of indicated, contraindicated and off-label use

In determining whether a prescription is off-label for a patient, we use two criteria to construct variables of interest: narrow and broad. The narrow criterion assesses if the specific medical condition linked to the prescription in the MEPS Conditions File matches an approved indication for the drug according to DrugCentral. The broad criterion, on the other hand, considers any medical condition recorded for the patient in the MEPS Conditions File to match an approved indication for the drug. A prescription is considered contraindicated if any medical condition recorded for the patient in the MEPS Conditions File is a contraindication for the drug according to DrugCentral. Finally, we construct one indicator that we set to 1 if the broad criterion is met and the prescription is not contraindicated; otherwise, we set this indicator to 0.

### 2.5. Statistical analysis

We examine the associations between off-label drug use, contraindicated drug use, and numerous measures of health status and health care utilisation, accounting for a rich set of confounders. Health status includes for example whether the person is unable to work or has (self-reported) activity or cognitive limitations. We control for a comprehensive set of individual attributes including whether the person has each of 477 medical conditions, the person’s sex, age, education, race, and calendar year (details are provided in the Supplemental Material). Since we study only patients who have already received prescriptions, our results are not confounded by barriers to accessing medications (for example geographic or health insurance), allowing us to focus on the relationship between prescribing patterns and health outcomes.

We use person-level data to analyse the association between off-label and contraindicated drug use, health status, and health care utilisation, controlling for a number of attributes of the person. Our primary objective is to examine the relationship between the fractions of the person’s prescriptions that were off-label and contraindicated and his or her health status and health care utilisation. We control in a very general way for the person’s number of prescriptions and for the mean FDA approval year of those prescriptions. We also control for whether the person has each of 477 medical conditions and for the person’s sex, single year of age and education, race and calendar year. We estimate general linear and probit regression models. To assess the robustness of our estimates with respect to omitted variable bias, we estimate associations based on a double machine learning approach using random forests and regression trees as learners (Chernozhukov et al., 2018).

## 3. Results

**Figure 1.**
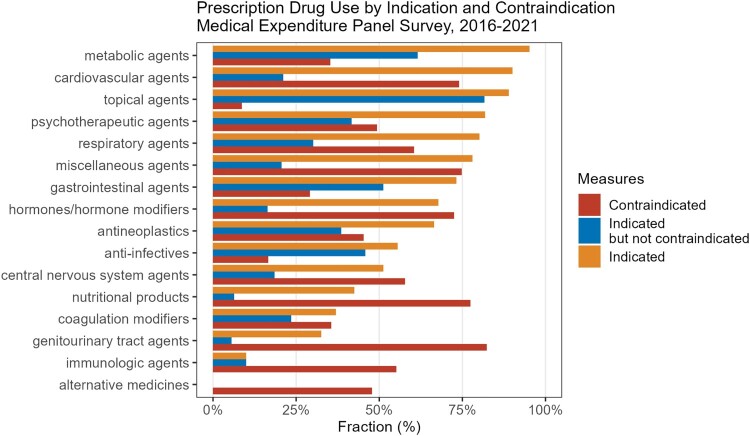
Prescription drug use by indication and contraindication. Notes: The figure shows the fractions of indicated, contraindicated and indicated but not contraindicated prescription drug uses according to indication / contraindication drug pairs in the US non-institutionalised population, 2015–2021. Therapeutic classes are ordered by number of prescriptions. Data was obtained from the Medical Expenditure Panel Surveys 2016–2021, DrugCentral.org and the SNOMED classification.

The analysis sample included 1.6 million prescriptions. Table 1 presents frequency estimates of indicated and contraindicated prescriptions at the prescription level. Figure 1 presents the fractions of indicated, contraindicated use as well as uses that were indicated, but not contraindicated by major therapeutic areas. Under a narrow definition of indication, only 56% of prescriptions were for labelled indications. Broadening the definition, 75% of prescriptions were for labelled indications, with 25% being off-label. Notably, this off-label proportion aligns closely with the average from three cited studies: 21% (Radley et al., 2006); 11% (Eguale et al., 2016); 29.9–38.3% (Bradford et al., 2018).

**Table 1.** Fraction of prescriptions that were indicated and contraindicated at prescription drug-level in the non-institutionalised American population.

Variable	Category	*N*	indic_narrow	indic_broad	contraindicated	indic_not_contra
All	–	1,655,101	56.5	75.09	54.34	32.92
Sex	male	697,174	56.72	75.17	53.14	33.73
	female	957,927	56.33	75.03	55.2	32.33
Education	missing	11,158	54.48	72.52	40.9	40.95
	0–11 years	333,066	55.22	74.92	54.46	32.55
	12 years	517,288	56.15	75.12	56.03	31.45
	13+ years	107,486	56.03	75.25	56.71	32.01
	18+ years	666,574	57.52	75.18	53.3	33.9
Age group	0–17	39,628	56.43	73.6	20.03	58.11
	18–44	278,849	54.3	71.31	46.02	37.76
	45–64	702,388	56.85	75.49	55.53	32.48
	65–84	568,400	57.5	76.79	58.46	30.13
	85 or older	60,475	52.92	72.3	57.27	29.11
Race	White	1,256,808	56.22	74.86	54.45	32.83
	Black	272,257	57.62	75.79	54.85	32.18
	American Indian-Alaska	15,348	51.88	74.71	58.18	29.91
	Asian	51,263	61.82	77.7	46.35	39.98
	Multiple	59,425	54.09	74.7	55.33	32.91
Year	2016	288,469	56.72	72.76	47.37	37.42
	2017	291,290	54.29	71.15	46.86	36.83
	2018	297,588	54.51	75.61	57.55	30.54
	2019	265,693	55.73	76.41	59.44	29.58
	2020	251,225	57.9	77.06	58.73	30.46
	2021	260,836	60.36	77.97	56.68	32.42
FDA approval year group	1941–1978	424,017	42.13	66.95	64.56	20.96
	1979–1991	381,966	67.09	83.78	55.67	35.42
	1992–1997	431,967	60.58	76.57	53.79	33.64
	1998–2018	388,135	57.23	73.78	42.46	42.71
Medicare	Medicare	534,965	56.72	76.07	58.61	29.92
	No Medicare	1,091,120	56.39	74.61	52.24	34.38
Medicaid	Medicaid	279,757	54.11	74.99	52.76	34.06
	No Medicaid	1,346,328	56.99	75.11	54.66	32.68

Notes: indic_narrow includes the specific medical condition linked to the prescription in the MEPS Conditions File matches an approved indication for the drug according to DrugCentral . indic_broad considers any medical condition recorded for the patient in the MEPS Conditions File to match an approved indication for the drug. Indic_not_contra captures if the broad definition of indicated use is met and the prescription is not contraindicated; *N* = number of prescriptions. Data was obtained from the Medical Expenditure Panel Surveys 2016–2021, DrugCentral.org and the SNOMED classification.

Calculations show that 54% of prescriptions were contraindicated, which means that patients had at least one medical condition for which the prescribed drug was not recommended. Only 33% of prescriptions met both the broad indication criteria and were not contraindicated. Contrary to expectations, contraindications were not more prevalent among off-label prescriptions compared to indicated ones. 48.8% of off-label prescriptions were contraindicated, whereas 56.2% of indicated prescriptions had contraindications.

Table 1 additionally illustrates fractions of indicated and contraindicated prescriptions across various demographic categories and across time. We find similar fractions of indicated and contraindicated prescriptions across genders, educational levels, and races. Across age groups, we find that the fractions of indicated prescriptions vary marginally, with higher ages correlating with more contraindications due to increased comorbidities. We observe higher contraindication rates during 2018–2021 compared to 2016–2017.

We observe similar fractions of indicated and contraindicated prescriptions between Medicaid and non-Medicaid categories, similar to Bradford et al. (2018). Prescriptions for individuals with Medicare coverage exhibit higher contraindication rates compared to non-Medicare, possibly due to the older age and higher comorbidity burden of Medicare patients. Finally, we observe different indication and contraindication rates by the FDA approval year, with older drugs more likely to be contraindicated.

Considering drug use by different Multum Therapeutic Classes, we see large variations in indicated, contraindicated and, indicated but not contraindicated use (Figure 1). Metabolic agents show the highest indicated use rate at approximately 95% of prescriptions, followed by cardiovascular agents (90% indicated use) and topical agents (88% indicated use). These three classes also show the highest rate of optimal prescribing expressed by indicated but not contraindicated use (30–40% of drug-disease combinations). Nevertheless, some therapeutic classes with high indicated use rates (like metabolic and cardiovascular agents) still show substantial contraindication rates, suggesting that even when drugs are used for approved indications, they may still pose risks for patients with certain conditions. Of note is also suboptimal use of drugs in genitourinary tract agents, where indicated use rates were 32%, contraindicated use was very high at 82% and optimal drug use was only 5%.

Table 2 shows the mean values and differences in health care use and health status by indicated and contraindicated drug use. The results indicate significant differences in health care use and health status indicators between individuals with indicated and contraindicated prescription drug use below and above the median. Individuals with higher indicated use generally have lower health care utilisation and better health status compared to those with lower indicated use. Conversely, individuals with higher contraindicated use show increased health care utilisation and worse health status compared to those with lower contraindicated use. All differences are statistically significant with *p*-values less than 0.0001.

**Table 2.** Health care use and health status by indicated (broad definition) and contraindicated prescription drug use.

Category	Variable	Mean (Indicated use < median)	Mean (Indicated use > median)	difference	*p*-value	Mean (Contraindicate use < median)	Mean (Contraindicated > median)	difference	*p*-value
Health care use	ztotexp	7.998	7.901	0.09742	<0.0001	7.43	8.469	−1.038	<0.0001
	ipdis	0.1721	0.1278	0.0443	<0.0001	0.08493	0.215	−0.1301	<0.0001
	ertot	0.3692	0.2892	0.08001	<0.0001	0.2446	0.4139	−0.1693	<0.0001
	obtotv	9.576	8.716	0.8595	<0.0001	6.459	11.83	−5.374	<0.0001
Health status	ANYLMT	0.3439	0.2982	0.04567	<0.0001	0.1877	0.4544	−0.2668	<0.0001
	ACTLIM31	0.1787	0.1369	0.04178	<0.0001	0.07548	0.2401	−0.1646	<0.0001
	UNABLE31	0.1187	0.08925	0.02942	<0.0001	0.04567	0.1622	−0.1166	<0.0001
	WLKLIM31	0.2257	0.1881	0.03763	<0.0001	0.1029	0.3109	−0.208	<0.0001
	WRKLIM31	0.1651	0.1237	0.04144	<0.0001	0.06868	0.2202	−0.1515	<0.0001
	SCHLIM31	0.07778	0.0573	0.02048	<0.0001	0.0304	0.1047	−0.07428	<0.0001
	HSELIM31	0.1129	0.08471	0.02818	<0.0001	0.04315	0.1545	−0.1113	<0.0001
	SOCLIM31	0.1006	0.07907	0.02155	<0.0001	0.0434	0.1363	−0.09288	<0.0001
	COGLIM31	0.09557	0.07526	0.02031	<0.0001	0.04152	0.1293	−0.08779	<0.0001

Notes: ztotexpend: Total health care expenditure (log); IPDIS: Number of hospital inpatient discharges; ERTOT: Total number of emergency room visits; OBTOTV: Number of office-based provider visits. ANYLMT: = 1 if respondent has any activity limitations, = 0 otherwise; ACTLIM31: = 1 if the respondent has activity limitations lasting 31 days or more, = 0 otherwise; UNABLE31: = 1 if the respondent has been unable to perform activities for 31 days or more; = 0 otherwise; WLKLIM31: = 1 if the respondent has limitations in walking lasting 31 days or more, = 0 otherwise; WRKLIM31: = 1 if the respondent has work limitations lasting 31 days or more, = 0 otherwise; SCHLIM31: = 1 if the respondent has school limitations lasting 31 days or more, = 0 otherwise; HSELIM31: = 1 if the respondent has home limitations lasting 31 days or more, = 0 otherwise; SOCLIM31: = 1 if the respondent has social activity limitations lasting 31 days or more, = 0 otherwise; COGLIM31: = 1 if the respondent has cognitive limitations lasting 31 days or more, = 0 otherwise. We used the broad definition of indicated prescription drug use. Data was obtained from the Medical Expenditure Panel Surveys 2016–2021, DrugCentral.org and the SNOMED classification.

Table 3 presents estimates of drug attribute coefficients from general linear model models of health status measures at the person level. The coefficients indicate significant relationships between prescription attributes, such as indication and contraindication, and health status. For instance, using a drug based on its labelled indication was associated with a -0.034 [95% confidence interval (CI): -0.0453; -0.0224] reduction in reporting any limitation, while using a contraindicated prescription drug was associated with an increase in reporting any limitation by 0.0369 [95% CI: 0.0231; 0.0501]. Estimated associations of indicated use with other outcomes related to health status were similar or smaller.

**Table 3.** Estimates of contraindicated and indicated prescription medicine use and health status, general linear regressions.

Outcome	Variable	Estimate	Std. Error	t value	*p*-value	95% CI high	95% CI low	Mean percentage
ANYLMT	contraindicated	0.0369	0.0070	5.2660	<0.0001	0.0232	0.0506	11.49%
	indicated	−0.0339	0.0059	−5.7910	<0.0001	−0.0454	−0.0224	−10.56%
ACTLIM31	contraindicated	0.0134	0.0057	2.3560	0.0185	0.0023	0.0246	8.51%
	indicated	−0.0289	0.0048	−6.0700	<0.0001	−0.0382	−0.0196	−18.30%
UNABLE31	contraindicated	0.0025	0.0049	0.5145	0.6069	−0.0071	0.0122	2.43%
	indicated	−0.0226	0.0041	−5.5074	<0.0001	−0.0307	−0.0146	−21.76%
WLKLIM31	contraindicated	0.0100	0.0062	1.5950	0.1106	−0.0023	0.0222	4.82%
	indicated	−0.0285	0.0052	−5.4670	<0.0001	−0.0388	−0.0183	−13.79%
WRKLIM31	contraindicated	0.0122	0.0056	2.1980	0.0280	0.0013	0.0231	8.44%
	indicated	−0.0276	0.0046	−5.9630	<0.0001	−0.0367	−0.0186	−19.14%
SCHLIM31	contraindicated	0.0013	0.0042	0.2994	0.7646	−0.0070	0.0096	1.88%
	indicated	−0.0149	0.0035	−4.2067	<0.0001	−0.0218	−0.0080	−22.04%
HSELIM31	contraindicated	−0.0023	0.0048	−0.4813	0.6303	−0.0118	0.0072	−2.36%
	indicated	−0.0201	0.0040	−4.9819	<0.0001	−0.0280	−0.0122	−20.37%
SOCLIM31	contraindicated	0.0104	0.0048	2.1560	0.0311	0.0009	0.0198	11.53%
	indicated	−0.0081	0.0040	−2.0210	0.0433	−0.0160	−0.0002	−9.02%
COGLIM31	contraindicated	0.0032	0.0046	0.7007	0.4835	−0.0058	0.0123	3.78%
	indicated	−0.0149	0.0039	−3.8691	0.0001	−0.0225	−0.0074	−17.45%

Notes: Estimates obtained from general linear models controlling for mean FDA approval year of prescribed drug, education, sex, age, race, year of MEPS survey, number of prescriptions and 477 medical conditions. ANYLMT: = 1 if respondent has any activity limitations, = 0 otherwise; ACTLIM31: = 1 if the respondent has activity limitations lasting 31 days or more, = 0 otherwise; UNABLE31: = 1 if the respondent has been unable to perform activities for 31 days or more; = 0 otherwise; WLKLIM31: = 1 if the respondent has limitations in walking lasting 31 days or more, = 0 otherwise; WRKLIM31: = 1 if the respondent has work limitations lasting 31 days or more, = 0 otherwise; SCHLIM31: = 1 if the respondent has school limitations lasting 31 days or more, = 0 otherwise; HSELIM31: = 1 if the respondent has home limitations lasting 31 days or more, = 0 otherwise; SOCLIM31: = 1 if the respondent has social activity limitations lasting 31 days or more, = 0 otherwise; COGLIM31: = 1 if the respondent has cognitive limitations lasting 31 days or more, = 0 otherwise. We used the broad definition of indicated prescription drug use. Data was obtained from the Medical Expenditure Panel Surveys 2016–2021, DrugCentral.org and the SNOMED classification.

Similarly, Table 4 presents coefficients from linear models of health care utilisation measures, highlighting the impact of prescription attributes on medical expenditure and utilisation patterns. We find consistently across these outcome measures that contraindicated use of medicines was associated with increased health care utilisation, albeit only significant for office-based visits. For example, health care expenditure was 3.38 percentage points (pp) higher [95% CI: −1.1 pp; 7.86 pp]. Indicated use of a medicine was associated with decreased health care utilisation. For example, health care expenditures were 11.7 percentage points lower [95% CI: −15.5 pp; −8 pp].

**Table 4.** Estimates of contraindicated and indicated prescription medicine use and health care use, general linear regressions.

Outcome	Variable	Estimate	Std. Error	*t* value	*p*-value	95% CI high	95% CI low	Mean percentage
HC expenditure (log)	contraindicated	0.0338	0.0229	1.4796	0.1390	−0.0110	0.0787	0.43%
	indicated	−0.1174	0.0191	−6.1484	<0.0001	−0.1549	−0.0800	−1.48%
Inpatient discharges	contraindicated	−0.0080	0.0085	−0.9454	0.3445	−0.0247	0.0086	−5.36%
	indicated	−0.0267	0.0071	−3.7599	0.0002	−0.0406	−0.0128	−17.80%
Emergency room visits	contraindicated	−0.0010	0.0138	−0.0755	0.9398	−0.0280	0.0259	−0.32%
	indicated	−0.0441	0.0115	−3.8388	0.0001	−0.0667	−0.0216	−13.40%
Office based visits	contraindicated	0.5972	0.2377	2.5127	0.0120	0.1313	1.0630	6.53%
	indicated	0.1736	0.1985	0.8746	0.3818	−0.2154	0.5626	1.90%

Notes: Estimates obtained from general linear models controlling for mean FDA approval year of prescribed drug, education, sex, age, race, year of MEPS survey, number of prescriptions and 477 medical conditions. We used the broad definition of indicated prescription drug use. Data was obtained from the Medical Expenditure Panel Surveys 2016–2021, DrugCentral.org and the SNOMED classification.

Comparing our estimates of the general linear model with estimates from probit regressions and the double machine learning approach (Supplemental Material), we find similar results with respect to magnitude and direction of the estimates. The exception is the estimate for the number of office-based visits that was positive in the general linear model, but negative and significant in the double machine learning estimates.

## 4. Discussion

In this study, we measured the prevalence of off-label and contraindicated prescription drug use in the American non-institutionalised population. Applying a broad definition of indicated use, 25% of prescription uses were off-label. 54% were contraindicated. Only 33% of uses were indicated but not contraindicated. While our measures of off-label use align with the estimates from physician-based prescription data, we are not aware of a comparable estimate of contraindicated use in the American population. We find considerable disadvantages from off-label and contraindicated use of prescription drugs. Using prescription drugs off-label or when indications are contraindicated was associated with worse health status and higher health care utilisation.

While off-label and contraindicated drug use does not vary across several observed demographic categories (gender, race, education), we find variations across age groups, by FDA approval year and over time (2016–2021). Potential causes for these variations could be advancements in clinical guidelines and regulatory measures like rational prescribing in pediatrics (Committee on Drugs et al., 2014). Observing higher off-label use rates in older compared to newer drugs could relate to the extensive clinical experience, broader mechanisms of action, cost considerations and regulatory flexibility of health insurance schemes in prescribing newer compared to older drugs (Bradford et al., 2018). As we find that patient outcomes and health care use are negatively associated with off-label and contraindicated use, more careful monitoring or regulations in rational prescribing could be options to minimise harm, but should consider keeping off-label use as an option when standard treatments fail.

Our results indicate that individuals considerably deviate from rational choices when using prescription drugs, and this may make them worse off given the negative associations with health status and health care cost we calculated. Pharmaceutical policies are generally based on the assumption that involved stakeholders make rational decisions (Vandenplas et al., 2022). Behavioural economics principles suggest that this is not always the case as people deviate from rational behaviour in rather predictable patterns (Kahneman, 2003). Our rationality, willpower, and self-interest may be bounded and influenced by several external factors.

Some of our findings are at odds with those of previous studies. Bradford et al. (2018, p. 867) found that ‘of those with insurance, those with Medicaid are the most likely to be prescribed off-label,’ but we observe virtually no difference between the off-label percentages of Medicaid and non-Medicaid prescriptions. Eguale et al. (2016) found that women received more off-label prescriptions than men, but we observe virtually no difference between the off-label percentages of female and male prescriptions. Eguale et al. (2016) also found that ‘sicker patients were less likely to receive off-label drugs, which may be the result of their poor health creating less room to “experiment” with a drug,’ but we find that patients with higher percentages of off-label prescriptions had worse health status and higher health care utilisation, controlling for many factors.

This study has limitations. While DrugCentral.org covers 2375 medicines described as chemical structures, we observe 348 medicines described as active ingredients which are self-administered by patients in the MEPS. In 2021, MEPS drug expenditure was 90% of overall pharmaceutical expenditures in the US (Tichy et al., 2023). Nevertheless, our measures of off-label and contraindicated uses may exclude drug-indication/contraindication pairs that are exclusively administered by health care providers not captured by self-administered data or which are rarely provided. Our measures of off-label and contraindicated use rely on self-reported data and may underreport the extent of off-label drug use in the MEPS which utilises pharmacy follow-backs to validate prescription drug use. For some conditions, especially mental illnesses, individuals may not report their condition or prescription in a survey because of a fear of stigma of suffering from that illness within the society. Self-reports have shown to deviate from administrative data and underreporting was higher in mental illnesses compared to cardiovascular diseases or diabetes (Bharadwaj et al., 2017). In addition, individuals may suffer from recall bias when reporting prescription medications (Hunger et al., 2013).

Our study faces limitations in establishing causal relationships between off-label/contraindicated use and health outcomes. While a randomised controlled trial would be ideal for causal inference, it would be neither feasible nor ethical to randomise patients to off-label drug use given existing effective on-label alternatives. Instead, we employ observational data and control for a comprehensive set of potential confounders, including detailed medical conditions, prescription patterns, and drug characteristics, using double-machine learning approaches to test the robustness of our estimates. Our results remain susceptible to self-selection bias, as unobservable factors – particularly disease severity and provider/patient decision-making processes – may influence both the likelihood of off-label/contraindicated prescribing and health outcomes. Our estimates should therefore be interpreted as robust conditional correlations rather than causal effects. Finally, while our study cannot observe individual provider characteristics and prescribing preferences, these unobserved factors are unlikely to substantially bias our findings since the same providers contribute to both indicated and off-label/contraindicated prescriptions for the same patient, effectively differencing out provider-specific effects.

## 5. Conclusions

This study of outpatient prescription drug use reveals that off-label and contraindicated use are associated with worse health status and inefficiencies in health care utilisation. Patients frequently use drugs that are unlikely to help them and may even harm them. Our measures of off-label and contraindicated use provide options to investigate the causes of high contraindicated drug use and the role of regulatory or educational interventions in reducing off-label and contraindicated prescriptions. By identifying these patterns, the study highlights potential areas for cost reduction in the healthcare system through better prescribing practices through strengthening collaboration between regulators, researchers and clinicians or clinical guidelines that use high-quality evidence, real-world data and expert knowledge to evaluate evidence about off-label use and appropriate prescribing.

## Supplementary Material

20240420 SupplementaryMaterial.docx

## Data Availability

The source data is publicly available from https://meps.ahrq.gov/mepsweb/data_stats/download_data_files.jsp and https://drugcentral.org/download. A data availability and provenance statement that includes the analysis data and analysis codes is available from https://osf.io/pzwg6/.

## Author description of the AI-generated content

ChatGPT 4.0 and earlier versions and Claude 3.5 Sonnet were used to assist with manuscript editing and revision, including drafting the revision response letter, developing structures for manuscript sections, analysing data visualisations, developing and modifying analysis code, and refining methodological explanations. Additionally, these tools helped with editing and rephrasing existing content to improve clarity. After using these tools, the authors reviewed and edited all content and take full responsibility for the content of the published article.

## Permission to reproduce material from other sources

No material was directly reproduced from other sources. The Medical Expenditure Panel survey is free for use without specific permission. DrugCentral.org is available under creative commons license (CC BY-SA 4.0).
