# Supplementary material for: Prevalence and relationship with health of off-label and contraindicated drug use in the United States: a cross-sectional study
Source: J Pharm Policy Pract. 2025 Mar 6;18(1):2472221. doi: 10.1080/20523211.2025.2472221 (PMC11892052; doi:10.1080/20523211.2025.2472221)
Supplement: 20240420 SupplementaryMaterial.docx [file JPPP_A_2472221_SM1121.docx]

OFF-LABEL AND CONTRAINDICATED DRUG USE: PREVALENCE AND RELATIONSHIP WITH HEALTH

Supplementary material

2024-07-10

# 1. Problem formulation

In the off-label use study, we are interested in the treatment effect of using drugs that are considered off-label or contraindicated on health care use and disability measures. We compare individuals who use off-label or contraindicated drugs (treatments) to individuals who use indicated drugs (controls). The ideal research design could randomize on-label and off-label use of available treatments by condition and individual. It would likely not be feasible to randomize off-label use for any individual in a controlled study. Based on the [Declaration of Helsinki](https://www.wma.net/policies-post/wma-declaration-of-helsinki-ethical-principles-for-medical-research-involving-human-subjects/) there must be scientific and compelling arguments to test an effective on-label use against a potentially less effective off-label use of a drug.

In our setting, the treatment variable is off-label or contraindicated prescription drug use by a patient. We cannot observe who made the decision to use the drug outside it’s indicated use, the patient or the provider. The decision is also likely affected by unobservable effects. For example, more severely ill patients may resort to off-label use. If we do not account for this type of self-selection, the estimated effect is likely to be biased.

We aim to establish conditional exogeneity of the effect of using off-label or contraindicated drugs by considering a rich set of possible confounders that simultaneously impact health status and health care utilization ([Figure 1](#fig-DAG)). We control in a very general way for the person’s number of prescriptions and for the mean FDA approval year of those prescriptions variables as follows: $x_{1}$: Number of prescriptions (RXs) and $x_{2}$: mean FDA approval year. These variables consider that a higher number of prescriptions increases the risk of drug-drug interactions and adverse events and that off-label and contraindicated drug use is more prevalent in older compared to newer drugs (Bradford, Turner, and Williams 2018).

We also control for whether the person has each of 477 medical conditions ($x_{3}$: Medical conditions), for the person’s sex ($x_{4}$), age ($x_{5}$), education ($x_{6}$), race ($x_{7}$, captured by 6 dimensions), and calendar year ($x_{8}$, captured by 6 dimensions).

| 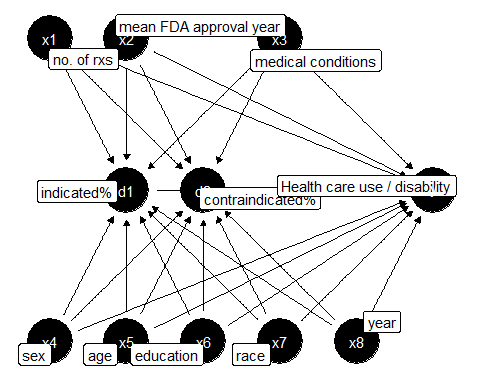  Figure 1: Directed Acyclic Graph of Health Care Utilization, off-label and contraindicated drug using, considering confounding factors |
| --- |

We aim to identify the average treatment effect of using an off-label (*=treatment*) compared to an on-label drug on health care use and disability (*=outcomes*). For our empirical estimates. Equation (1) specifies a causal model that identifies a partially linear treatment effect of off-label and contraindicated use:

$Y_{i}=d_{i}\theta_{0}+g_{0}\left( X_{i} \right)+\zeta_{i}$ (1)

where $Y_{i}$ represents the set of outcomes related to health status and health care use for individual $i$; $d_{i}$ represents the treatment variables for off-label and contraindicated use. The function $g_{0}\left( X_{i} \right)$ includes the high-dimensional vector $X=\left( X_{1},...,X_{p} \right)$ of confounding covariates. $\zeta_{i}$ denotes the stochastic error.

$\zeta_{i}\sim N\left( 0,1 \right)$ (2)

We assume that our treatment variables are also a function of the high-dimensional vector of confounding variables $m_{0}\left( X_{i} \right)$:

$d_{i}=m_{0}\left( X_{i} \right)+v_{i}$ (3)

where $v_{i}$ is the stochastic error and $v_{i}\sim N\left( 0,1 \right)$.

where $Y_{i}$ represents the set of outcomes related to health status and health care use for individual $i$; $d_{i}$ represents the treatment variables for off-label and contraindicated use. The function $g_{0}\left( X_{i} \right)$ includes the high-dimensional vector $X=\left( X_{1},...,X_{p} \right)$ of confounding covariates. $\zeta_{i}$ denotes the stochastic error.

$v_{i}\sim N\left( 0,1 \right)$ (4)

In a first specification, we estimate general linear models where we assume that the functional form of $g_{0}\left( X_{i} \right)$ and $m_{0}\left( X_{i} \right)$ is linear. In a second set of analyses, we employ Double Machine Learning (DML) to minimize relevant sources of potential biases. The most important concern is that we cannot adequately capture disease severity which is not directly observable in the MEPS data which could induce omitted variable bias. Disease severity can affect both the treatment of receiving off-label drugs and the outcomes related to health care use and health status.

The DML leverages machine learning algorithms to model the relationship between covariates, treatment and outcome using a wide range of covariates, ensuring that the effect of omitted variables is minimized (Chernozhukov et al. 2018). The MEPS data allows to control for 477 medical conditions as covariates. In addition to minimizing omitted variable bias, DML also minimizes that the functional form is incorrectly specified. The general linear models assume that all covariates are considered to have a linear relationship with both the treatment and the outcome. The DML algorithms used are more flexible. Another concern that comes along with controlling for a high number of covariates is overfitting bias such that the generalization of the estimates to new data may be limited. DML uses training and test data sets to avoid overfitting. To balance overfitting and regularization with regularization penalizing model complexity to avoiding overfitting, the DML aims to balance regularization through cross-fitting. Comparing the estimates of the GLM and the DML allows us assessing the extent of bias when assuming a simple linear functional form in the GLM.

# 2. Measures and data

## 2.1 Measures of off-label and contraindicated use

A prescription is off-label if it is not indicated for the patient. There are two different ways—narrow and broad—to determine whether a MEPS prescription is indicated. Our two alternative criteria for determining whether prescription $p$ for person $i$ was indicated, and our criterion for determining whether it was contraindicated, are:

$indic\text{-}narrow_{pi}=1$ (5)

if the 3-digit ICD10 code in the MEPS Conditions File record to which prescription $p$ for drug $d$ for person $i$ is linked (by the CLNK file) is an approved indication of drug $d$, according to DrugCentral; $=0$ otherwise.

$indic\text{-}broad_{pi}=1$ (6)

if a 3-digit ICD10 code in any of person $i$’s records in the MEPS Conditions File is an approved indication of drug $d$, according to DrugCentral; $=0$ otherwise.

$contraindic_{pi}=1$ (7)

if a 3-digit ICD10 code in any of person $i$’s records in the MEPS Conditions File is a contraindication of drug $d$, according to DrugCentral; $=0$ otherwise.

We also define

${indic\_not\_contra}_{pi}=1$ (8)

if $indic\text{-}broad_{pi}=1$ and $contraindic_{pi}=0$, and $=0$ otherwise.

## 2.2 Sample specification

We exclude individuals that do not report any use of prescription drugs such that the final analysis sample includes $46,770$ individuals covering $1,596,753$ prescriptions.

# 3. Empirical analysis

## 3.1 Descriptive statistics

### 3.1.1 Variables of interest and person level characteristics

Table 1 shows the percentage of prescription drugs used by individuals in the MEPS that are indicated based on the broad and narrow definition (first two columns) and the share contraindicated (third column) and prescriptions that are indicated but not contraindicated (fourth column). The data reported here are aggregated at the person level.

Table 1: Fraction of prescriptions that were indicated and contraindicated - person-level estimates

| **variable** | **category** | **N** | **indic_broad** | **indic_narrow** | **contraindicated** | **indic_not_contra** |
| --- | --- | --- | --- | --- | --- | --- |
| All | 1 | 46770 | 55.38 | 67.08 | 33.36 | 42.66 |
| SEX | 1 | 20169 | 56.06 | 67.64 | 31.94 | 43.84 |
| SEX | 2 | 26601 | 54.86 | 66.66 | 34.44 | 41.77 |
| educat | missing | 756 | 54.32 | 66.07 | 14.79 | 55.85 |
| educat | 0-11 years | 8927 | 53.6 | 65.79 | 31.46 | 42.81 |
| educat | 12 years | 12409 | 55.4 | 67.48 | 37.3 | 40.05 |
| educat | 13+ years | 2688 | 54.95 | 67.1 | 37.09 | 40.5 |
| educat | 18+ years | 20199 | 56.56 | 67.73 | 33.95 | 42.83 |
| agecat | 0-17 | 4488 | 51.52 | 62.14 | 9.09 | 55.94 |
| agecat | 18-44 | 12357 | 48.75 | 58.24 | 23.23 | 43.49 |
| agecat | 45-64 | 16078 | 57.7 | 69.58 | 37.65 | 41.76 |
| agecat | 65-84 | 11953 | 60.52 | 74.52 | 46.2 | 38.45 |
| agecat | 85 or older | 1442 | 55.18 | 69.51 | 47.04 | 35.66 |
| racecat | White | 34774 | 55.25 | 67.1 | 33.66 | 42.54 |
| racecat | Black | 7727 | 56.54 | 67.79 | 34.21 | 42.35 |
| racecat | Amer Indian-Alaska | 381 | 50.08 | 61.63 | 35.16 | 36.78 |
| racecat | Asian | 2377 | 55.98 | 66.07 | 27.42 | 45.54 |
| racecat | Multiple | 1511 | 52.77 | 66.02 | 31.01 | 44.18 |
| yearcat | 2016 | 17600 | 52.26 | 63.55 | 27.66 | 44.08 |
| yearcat | 2017 | 8175 | 54.82 | 64.26 | 26.89 | 45.68 |
| yearcat | 2018 | 9985 | 55.22 | 70.43 | 41.52 | 39.08 |
| yearcat | 2019 | 4987 | 57.92 | 70.6 | 41.63 | 39.29 |
| yearcat | 2020 | 3020 | 62.53 | 72.85 | 39.3 | 42.47 |
| yearcat | 2021 | 3003 | 64.3 | 72.65 | 37.53 | 43.88 |
| FDAcat | 1941-1978 | 12013 | 40.15 | 50.94 | 23.73 | 37.11 |
| FDAcat | 1979-1991 | 20460 | 58.77 | 72.51 | 41.61 | 40.69 |
| FDAcat | 1992-1997 | 10431 | 63.95 | 74.54 | 33.77 | 48.11 |
| FDAcat | 1998-2018 | 3866 | 61.61 | 68.39 | 18.5 | 55.68 |
| medicarecat | No Medicare | 12931 | 58.77 | 74.53 | 51.07 | 34.9 |
| medicarecat | Medicare | 2659 | 60.67 | 71.52 | 36.93 | 44.32 |
| medicaidcat | No Medicaid | 6114 | 55.13 | 71.44 | 45.12 | 37.55 |
| medicaidcat | Medicaid | 5721 | 49.45 | 60.13 | 20.13 | 46.83 |
| Data sources: Medical Expenditure Panel Survey, 2015-2021, DrugCentral.Org and SNOMED | | | | | | |

### 3.1.2 Outcomes by off-label and contraindicated use

Table 2 shows the outcomes of health care use and health status by indicated drug use considering the broad definition of indicated use. Table 3 the outcomes of health care use and health status by indicated drug use considering the broad definition of indicated use. Table 4 shows the outcomes of health care use and health status by contraindicated use.

Table 2: Outcome variables by indicated use (broad)

|  | **0** | | **1** | |  | |
| --- | --- | --- | --- | --- | --- | --- |
|  | **Mean** | **Std. Dev.** | **Mean** | **Std. Dev.** | **Diff. in Means** | **Std. Error** |
| ztotexp | 7.6113 | 1.7076 | 8.0877 | 1.6151 | 0.4764*** | 0.0171 |
| ipdis | 0.1308 | 0.4548 | 0.1578 | 0.5300 | 0.0270*** | 0.0049 |
| ertot | 0.3162 | 0.8071 | 0.3345 | 0.8153 | 0.0183* | 0.0082 |
| obtotv | 7.5900 | 13.6378 | 9.7819 | 15.2264 | 2.1919*** | 0.1438 |
| ANYLMT | 0.2479 | 0.4318 | 0.3510 | 0.4773 | 0.1031*** | 0.0045 |
| ACTLIM31 | 0.1189 | 0.3237 | 0.1737 | 0.3788 | 0.0548*** | 0.0035 |
| UNABLE31 | 0.0757 | 0.2645 | 0.1155 | 0.3196 | 0.0398*** | 0.0029 |
| WLKLIM31 | 0.1495 | 0.3566 | 0.2303 | 0.4211 | 0.0808*** | 0.0038 |
| WRKLIM31 | 0.1103 | 0.3132 | 0.1584 | 0.3651 | 0.0481*** | 0.0034 |
| SCHLIM31 | 0.0515 | 0.2211 | 0.0741 | 0.2619 | 0.0226*** | 0.0024 |
| HSELIM31 | 0.0726 | 0.2595 | 0.1095 | 0.3123 | 0.0369*** | 0.0028 |
| SOCLIM31 | 0.0662 | 0.2486 | 0.0995 | 0.2993 | 0.0333*** | 0.0027 |
| COGLIM31 | 0.0646 | 0.2459 | 0.0939 | 0.2917 | 0.0293*** | 0.0026 |
| direct_binary | 0.0000 | 0.0000 | 0.7907 | 0.4068 | 0.7907*** | 0.0022 |
| contraindicated_binary | 0.1928 | 0.3945 | 0.3808 | 0.4856 | 0.1880*** | 0.0043 |
| Notes: Data sources: Medical Expenditure Panel Survey, 2015-2021, DrugCentral.Org and SNOMED ANYLMT: = 1 if respondent has any activity limitations, =0 otherwise; ACTLIM31: = 1 if the respondent has activity limitations lasting 31 days or more, = 0 otherwise; UNABLE31: = 1 if the respondent has been unable to perform activities for 31 days or more; = 0 otherwise; WLKLIM31: = 1 if the respondent has limitations in walking lasting 31 days or more, = 0 otherwise; WRKLIM31: = 1 if the respondent has work limitations lasting 31 days or more, = 0 otherwise; SCHLIM31: = 1 if the respondent has school limitations lasting 31 days or more, = 0 otherwise; HSELIM31: = 1 if the respondent has home limitations lasting 31 days or more, = 0 otherwise; SOCLIM31: = 1 if the respondent has social activity limitations lasting 31 days or more, = 0 otherwise; COGLIM31: = 1 if the respondent has cognitive limitations lasting 31 days or more, = 0 otherwise. ztotexpend: Total health care expenditure (log); IPDIS: Number of hospital inpatient discharges; ERTOT: Total number of emergency room visits; OBTOTV: Number of office-based provider visits. | | | | | | |

Table 3: Outcome variables by indicated use (narrow)

|  | **0** | | **1** | |  | |
| --- | --- | --- | --- | --- | --- | --- |
|  | **Mean** | **Std. Dev.** | **Mean** | **Std. Dev.** | **Diff. in Means** | **Std. Error** |
| ztotexp | 7.8858 | 1.7093 | 7.9994 | 1.6125 | 0.1136*** | 0.0155 |
| ipdis | 0.1667 | 0.5384 | 0.1369 | 0.4852 | -0.0299*** | 0.0048 |
| ertot | 0.3709 | 0.8798 | 0.2967 | 0.7550 | -0.0742*** | 0.0077 |
| obtotv | 9.1898 | 15.5645 | 9.1119 | 14.2047 | -0.0779 | 0.1396 |
| ANYLMT | 0.3209 | 0.4668 | 0.3212 | 0.4670 | 3.646e-04 | 0.0044 |
| ACTLIM31 | 0.1669 | 0.3729 | 0.1506 | 0.3577 | -0.0162*** | 0.0034 |
| UNABLE31 | 0.1112 | 0.3144 | 0.0983 | 0.2977 | -0.0130*** | 0.0029 |
| WLKLIM31 | 0.2095 | 0.4070 | 0.2048 | 0.4036 | -0.0047 | 0.0038 |
| WRKLIM31 | 0.1543 | 0.3612 | 0.1367 | 0.3436 | -0.0175*** | 0.0033 |
| SCHLIM31 | 0.0731 | 0.2602 | 0.0632 | 0.2434 | -0.0098*** | 0.0024 |
| HSELIM31 | 0.1036 | 0.3047 | 0.0951 | 0.2933 | -0.0085** | 0.0028 |
| SOCLIM31 | 0.0951 | 0.2933 | 0.0857 | 0.2800 | -0.0093*** | 0.0027 |
| COGLIM31 | 0.0908 | 0.2873 | 0.0812 | 0.2732 | -0.0096*** | 0.0026 |
| indicated_binary | 0.3388 | 0.4733 | 1.0000 | 0.0000 | 0.6612*** | 0.0033 |
| contraindicated_binary | 0.2911 | 0.4543 | 0.3538 | 0.4782 | 0.0627*** | 0.0043 |
| Notes: Data sources: Medical Expenditure Panel Survey, 2015-2021, DrugCentral.Org and SNOMED ANYLMT: = 1 if respondent has any activity limitations, =0 otherwise; ACTLIM31: = 1 if the respondent has activity limitations lasting 31 days or more, = 0 otherwise; UNABLE31: = 1 if the respondent has been unable to perform activities for 31 days or more; = 0 otherwise; WLKLIM31: = 1 if the respondent has limitations in walking lasting 31 days or more, = 0 otherwise; WRKLIM31: = 1 if the respondent has work limitations lasting 31 days or more, = 0 otherwise; SCHLIM31: = 1 if the respondent has school limitations lasting 31 days or more, = 0 otherwise; HSELIM31: = 1 if the respondent has home limitations lasting 31 days or more, = 0 otherwise; SOCLIM31: = 1 if the respondent has social activity limitations lasting 31 days or more, = 0 otherwise; COGLIM31: = 1 if the respondent has cognitive limitations lasting 31 days or more, = 0 otherwise. ztotexpend: Total health care expenditure (log); IPDIS: Number of hospital inpatient discharges; ERTOT: Total number of emergency room visits; OBTOTV: Number of office-based provider visits. | | | | | | |

Table 4: Outcome variables by contraindicated use

|  | **0** | | **1** | |  | |
| --- | --- | --- | --- | --- | --- | --- |
|  | **Mean** | **Std. Dev.** | **Mean** | **Std. Dev.** | **Diff. in Means** | **Std. Error** |
| ztotexp | 7.6682 | 1.6504 | 8.5305 | 1.5120 | 0.8624*** | 0.0154 |
| ipdis | 0.1101 | 0.4130 | 0.2324 | 0.6582 | 0.1223*** | 0.0058 |
| ertot | 0.2744 | 0.7345 | 0.4425 | 0.9447 | 0.1681*** | 0.0087 |
| obtotv | 7.4739 | 12.7783 | 12.5990 | 17.8320 | 5.1250*** | 0.1613 |
| ANYLMT | 0.2440 | 0.4295 | 0.4802 | 0.4996 | 0.2362*** | 0.0047 |
| ACTLIM31 | 0.1077 | 0.3100 | 0.2612 | 0.4393 | 0.1535*** | 0.0040 |
| UNABLE31 | 0.0681 | 0.2519 | 0.1780 | 0.3825 | 0.1099*** | 0.0034 |
| WLKLIM31 | 0.1451 | 0.3522 | 0.3345 | 0.4718 | 0.1895*** | 0.0043 |
| WRKLIM31 | 0.0983 | 0.2977 | 0.2398 | 0.4270 | 0.1415*** | 0.0038 |
| SCHLIM31 | 0.0440 | 0.2051 | 0.1161 | 0.3204 | 0.0721*** | 0.0028 |
| HSELIM31 | 0.0648 | 0.2461 | 0.1691 | 0.3748 | 0.1043*** | 0.0033 |
| SOCLIM31 | 0.0606 | 0.2385 | 0.1503 | 0.3574 | 0.0898*** | 0.0032 |
| COGLIM31 | 0.0584 | 0.2344 | 0.1413 | 0.3483 | 0.0829*** | 0.0031 |
| direct_binary | 0.5384 | 0.4985 | 0.6087 | 0.4881 | 0.0702*** | 0.0048 |
| indicated_binary | 0.6525 | 0.4762 | 0.8286 | 0.3769 | 0.1761*** | 0.0041 |
| Notes: Data sources: Medical Expenditure Panel Survey, 2015-2021, DrugCentral.Org and SNOMED ANYLMT: = 1 if respondent has any activity limitations, =0 otherwise; ACTLIM31: = 1 if the respondent has activity limitations lasting 31 days or more, = 0 otherwise; UNABLE31: = 1 if the respondent has been unable to perform activities for 31 days or more; = 0 otherwise; WLKLIM31: = 1 if the respondent has limitations in walking lasting 31 days or more, = 0 otherwise; WRKLIM31: = 1 if the respondent has work limitations lasting 31 days or more, = 0 otherwise; SCHLIM31: = 1 if the respondent has school limitations lasting 31 days or more, = 0 otherwise; HSELIM31: = 1 if the respondent has home limitations lasting 31 days or more, = 0 otherwise; SOCLIM31: = 1 if the respondent has social activity limitations lasting 31 days or more, = 0 otherwise; COGLIM31: = 1 if the respondent has cognitive limitations lasting 31 days or more, = 0 otherwise. ztotexpend: Total health care expenditure (log); IPDIS: Number of hospital inpatient discharges; ERTOT: Total number of emergency room visits; OBTOTV: Number of office-based provider visits. | | | | | | |

## 3.2 General linear regression estimates

Table 5 shows general linear regression estimates of health status and health care utilization, considering contraindicated, indicated and FDA approval year as explanatory variables.

Table 5: Estimates of health status and health care utilization and indicated or contraindicated prescription drug use, General Linear Regression estimates

| **outcome** | **Variable** | **Estimate** | **Std. Error** | **pvalue** | **95% CI – lower bound** | **95% CI – upper bound** | **Mean percentage** |
| --- | --- | --- | --- | --- | --- | --- | --- |
| ztotexp | contraindicated | 0.03384 | 0.02287 | 1.390e-01 | -0.01099 | 0.07867 | 0.43 |
| ztotexp | indicated | -0.1174 | 0.0191 | 7.889e-10 | -0.1549 | -0.08001 | -1.48 |
| ztotexp | year_fda | 0.0002856 | 0.0005944 | 6.308e-01 | -0.0008794 | 0.001451 | 0 |
| ipdis | contraindicated | -0.008035 | 0.008499 | 0.3444802 | -0.02469 | 0.008624 | -5.36 |
| ipdis | indicated | -0.02669 | 0.007098 | 0.0001702 | -0.0406 | -0.01278 | -17.8 |
| ipdis | year_fda | -0.0004428 | 0.0002209 | 0.0450218 | -0.0008757 | -0.000009827 | -0.3 |
| ertot | contraindicated | -0.001039 | 0.01376 | 9.398e-01 | -0.02801 | 0.02594 | -0.32 |
| ertot | indicated | -0.04412 | 0.01149 | 1.238e-04 | -0.06665 | -0.0216 | -13.4 |
| ertot | year_fda | -0.002033 | 0.0003577 | 1.327e-08 | -0.002734 | -0.001332 | -0.62 |
| obtotv | contraindicated | 0.5972 | 0.2377 | 0.011986 | 0.1313 | 1.063 | 6.53 |
| obtotv | indicated | 0.1736 | 0.1985 | 0.381795 | -0.2154 | 0.5626 | 1.9 |
| obtotv | year_fda | -0.01641 | 0.006176 | 0.007888 | -0.02852 | -0.004305 | -0.18 |
| ANYLMT | contraindicated | 0.0369 | 0.007008 | 1.401e-07 | 0.02317 | 0.05064 | 11.49 |
| ANYLMT | indicated | -0.03389 | 0.005853 | 7.042e-09 | -0.04537 | -0.02242 | -10.56 |
| ANYLMT | year_fda | -0.0005307 | 0.0001821 | 3.570e-03 | -0.0008877 | -0.0001737 | -0.17 |
| ACTLIM31 | contraindicated | 0.01342 | 0.005697 | 1.846e-02 | 0.002258 | 0.02459 | 8.51 |
| ACTLIM31 | indicated | -0.02888 | 0.004758 | 1.291e-09 | -0.0382 | -0.01955 | -18.3 |
| ACTLIM31 | year_fda | -0.0004097 | 0.000148 | 5.647e-03 | -0.0006999 | -0.0001196 | -0.26 |
| UNABLE31 | contraindicated | 0.00253 | 0.004918 | 6.069e-01 | -0.007109 | 0.01217 | 2.43 |
| UNABLE31 | indicated | -0.02262 | 0.004107 | 3.662e-08 | -0.03067 | -0.01457 | -21.76 |
| UNABLE31 | year_fda | -0.000255 | 0.0001278 | 4.606e-02 | -0.0005055 | -0.000004456 | -0.25 |
| WLKLIM31 | contraindicated | 0.00997 | 0.006249 | 1.106e-01 | -0.002278 | 0.02222 | 4.82 |
| WLKLIM31 | indicated | -0.02853 | 0.005219 | 4.612e-08 | -0.03876 | -0.0183 | -13.79 |
| WLKLIM31 | year_fda | -0.0004816 | 0.0001624 | 3.022e-03 | -0.0008 | -0.0001633 | -0.23 |
| WRKLIM31 | contraindicated | 0.0122 | 0.00555 | 2.799e-02 | 0.001318 | 0.02307 | 8.44 |
| WRKLIM31 | indicated | -0.02764 | 0.004635 | 2.497e-09 | -0.03673 | -0.01855 | -19.14 |
| WRKLIM31 | year_fda | -0.0004177 | 0.0001442 | 3.783e-03 | -0.0007004 | -0.000135 | -0.29 |
| SCHLIM31 | contraindicated | 0.001269 | 0.004237 | 7.646e-01 | -0.007036 | 0.009573 | 1.88 |
| SCHLIM31 | indicated | -0.01489 | 0.003539 | 2.596e-05 | -0.02182 | -0.00795 | -22.04 |
| SCHLIM31 | year_fda | -0.0002214 | 0.0001101 | 4.434e-02 | -0.0004373 | -0.000005602 | -0.33 |
| HSELIM31 | contraindicated | -0.002328 | 0.004838 | 6.303e-01 | -0.01181 | 0.007154 | -2.36 |
| HSELIM31 | indicated | -0.02013 | 0.00404 | 6.321e-07 | -0.02805 | -0.01221 | -20.37 |
| HSELIM31 | year_fda | -0.0001929 | 0.0001257 | 1.249e-01 | -0.0004393 | 0.00005351 | -0.2 |
| SOCLIM31 | contraindicated | 0.01036 | 0.004803 | 0.03105 | 0.0009437 | 0.01977 | 11.53 |
| SOCLIM31 | indicated | -0.008107 | 0.004012 | 0.04330 | -0.01597 | -0.0002441 | -9.02 |
| SOCLIM31 | year_fda | -0.0001356 | 0.0001248 | 0.27748 | -0.0003802 | 0.0001091 | -0.15 |
| COGLIM31 | contraindicated | 0.003232 | 0.004613 | 0.4835171 | -0.005809 | 0.01227 | 3.78 |
| COGLIM31 | indicated | -0.01491 | 0.003853 | 0.0001094 | -0.02246 | -0.007355 | -17.45 |
| COGLIM31 | year_fda | -0.0001281 | 0.0001199 | 0.2854600 | -0.000363 | 0.0001069 | -0.15 |

##

## 3.3 Probit regression results

Table 6 shows probit regression estimates of health status and health care utilization, considering contraindicated, indicated and FDA approval year as explanatory variables.

Table 6: Probit regression results

| **Model** | **Row** | **Parameter** | | **Estimate** |  | **Std Err** | **ChiSquare** | **Pr>Chi** |  | **abs change** | **% change** |
| --- | --- | --- | --- | --- | --- | --- | --- | --- | --- | --- | --- |
| 1 |  |  | **Dep. Var.: any ADL, IADL, functional, or activity limitations (ANYLMT; mean = 0.31)** | | | | | | | | |
|  | 1 | indic_broad | | **-0.133** |  | **0.025** | **27.44** | **<.0001** |  | -0.016 | -4.8% |
|  | 2 | contraindic | | **0.136** |  | **0.028** | **23.78** | **<.0001** |  | -0.016 | -4.9% |
|  | 3 | year_fda | | **-0.002** |  | **0.001** | **5.12** | **0.0237** |  | -0.006 | -2.0% |
|  |  |  | |  |  |  |  |  |  |  |  |
| 2 |  |  | **Dep. Var.: ANY LIMITATION WORK/HOUSEWORK/SCHOOL (ACTLIM; mean = 0.15)** | | | | | | | | |
|  | 4 | indic_broad | | **-0.213** |  | **0.032** | **43.43** | **<.0001** |  | -0.017 | -10.1% |
|  | 5 | contraindic | | **0.108** |  | **0.034** | **10.13** | **0.0015** |  | -0.009 | -5.3% |
|  | 6 | year_fda | | **-0.002** |  | **0.001** | **5.72** | **0.0167** |  | -0.006 | -3.6% |
|  |  |  | |  |  |  |  |  |  |  |  |
| 3 |  |  | **Dep. Var.: COMPLETELY UNABLE TO DO ACTIVITY (UNABLE; mean = 0.10)** | | | | | | | | |
|  | 7 | indic_broad | | **-0.248** |  | **0.038** | **41.93** | **<.0001** |  | -0.015 | -13.1% |
|  | 8 | contraindic | | 0.077 |  | 0.040 | 3.77 | 0.0522 |  | -0.005 | -4.3% |
|  | 9 | year_fda | | -0.001 |  | 0.001 | 1.39 | 0.2385 |  | -0.002 | -2.4% |
|  |  |  | |  |  |  |  |  |  |  |  |
| 4 |  |  | **Dep. Var.: LIMITATION IN PHYSICAL FUNCTIONING (WLKLIM; mean = 0.20)** | | | | | | | | |
|  | 10 | indic_broad | | **-0.158** |  | **0.030** | **27.74** | **<.0001** |  | -0.015 | -7.0% |
|  | 11 | contraindic | | **0.073** |  | **0.032** | **5.40** | **0.0201** |  | -0.007 | -3.3% |
|  | 12 | year_fda | | **-0.003** |  | **0.001** | **8.98** | **0.0027** |  | -0.008 | -3.7% |
|  |  |  | |  |  |  |  |  |  |  |  |
| 5 |  |  | **Dep. Var.: WORK LIMITATION (WRKLIM; mean = 0.14)** | | | | | | | | |
|  | 13 | indic_broad | | **-0.222** |  | **0.033** | **44.64** | **<.0001** |  | -0.017 | -10.8% |
|  | 14 | contraindic | | **0.112** |  | **0.035** | **10.24** | **0.0014** |  | -0.008 | -5.6% |
|  | 15 | year_fda | | **-0.003** |  | **0.001** | **6.14** | **0.0132** |  | -0.006 | -3.9% |
|  |  |  | |  |  |  |  |  |  |  |  |
| 6 |  |  | **Dep. Var.: SCHOOL LIMITATION (SCHLIM; mean =0.07)** | | | | | | | | |
|  | 16 | indic_broad | | **-0.217** |  | **0.042** | **26.84** | **<.0001** |  | -0.010 | -12.9% |
|  | 17 | contraindic | | 0.069 |  | 0.044 | 2.46 | 0.1168 |  | -0.003 | -4.3% |
|  | 18 | year_fda | | **-0.003** |  | **0.001** | **4.80** | **0.0285** |  | -0.004 | -5.3% |
|  |  |  | |  |  |  |  |  |  |  |  |
| 7 |  |  | **Dep. Var.: HOUSEWORK LIMITATION (HSELIM; mean = 0.10)** | | | | | | | | |
|  | 19 | indic_broad | | **-0.233** |  | **0.039** | **36.76** | **<.0001** |  | -0.014 | -12.6% |
|  | 20 | contraindic | | 0.043 |  | 0.040 | 1.15 | 0.2834 |  | -0.002 | -2.5% |
|  | 21 | year_fda | | -0.002 |  | 0.001 | 2.86 | 0.091 |  | -0.003 | -3.5% |
|  |  |  | |  |  |  |  |  |  |  |  |
| 8 |  |  | **Dep. Var.: SOCIAL LIMITATIONS (SOCLIM; mean = 0.09)** | | | | | | | | |
|  | 22 | indic_broad | | **-0.080** |  | **0.037** | **4.73** | **0.0297** |  | -0.004 | -4.7% |
|  | 23 | contraindic | | **0.118** |  | **0.039** | **9.28** | **0.0023** |  | -0.006 | -6.8% |
|  | 24 | year_fda | | -0.001 |  | 0.001 | 0.87 | 0.3522 |  | -0.002 | -1.8% |
|  |  |  | |  |  |  |  |  |  |  |  |
| 9 |  |  | **Dep. Var.: COGNITIVE LIMITATIONS (COGLIM; mean = 0.08)** | | | | | | | | |
|  | 25 | indic_broad | | **-0.153** |  | **0.038** | **16.04** | **<.0001** |  | -0.008 | -8.8% |
|  | 26 | contraindic | | 0.065 |  | 0.041 | 2.60 | 0.1066 |  | -0.003 | -3.9% |
|  | 27 | year_fda | | 0.000 |  | 0.001 | 0.02 | 0.8922 |  | 0.000 | -0.4% |

## 3.4 Double machine learning estimates

Both treatment and outcome are continuous variables such that we use regression learners. We select two types of learners for prediction:

1. **Random forest learner**: We followed (Probst, Wright, and Boulesteix (2019)) to set hyperparameters (mtry).
2. **Regression tree learner**

Table 7 shows the estimates of indicated and contraindicated use based on the double-machine learning estimates.

Table 7: Estimates of health status and health care utilization and indicated or contraindicated prescription drug use, Double Machine Learning estimates

| **outcome** | **learner** | **treatment** | **Estimate** | **Std. Error** | **95% CI – lower bound** | **95% CI – upper bound** | **p-value** | **Mean percentage** |
| --- | --- | --- | --- | --- | --- | --- | --- | --- |
| ACTLIM31 | forest | contraindicated | 0.0174 | 0.0058 | 0.00594 | 0.02886 | 0.0029 | 11.03 |
| ACTLIM31 | rpart | contraindicated | 0.0775 | 0.0055 | 0.06681 | 0.08829 | 0 | 49.15 |
| ACTLIM31 | forest | indicated | -0.0477 | 0.0043 | -0.05619 | -0.03929 | 0 | -30.26 |
| ACTLIM31 | rpart | indicated | -0.0527 | 0.0044 | -0.06125 | -0.04411 | 0 | -33.39 |
| ANYLMT | forest | contraindicated | 0.0371 | 0.0074 | 0.02256 | 0.05168 | 0 | 11.56 |
| ANYLMT | rpart | contraindicated | 0.1099 | 0.0069 | 0.09632 | 0.12353 | 0 | 34.24 |
| ANYLMT | forest | indicated | -0.0588 | 0.0057 | -0.06986 | -0.04766 | 0 | -18.30 |
| ANYLMT | rpart | indicated | -0.0632 | 0.0058 | -0.07453 | -0.05195 | 0 | -19.70 |
| COGLIM31 | forest | contraindicated | 0.006 | 0.0046 | -0.003038 | 0.01509 | 0.1926 | 7.05 |
| COGLIM31 | rpart | contraindicated | 0.0426 | 0.0044 | 0.03404 | 0.05110 | 0 | 49.84 |
| COGLIM31 | forest | indicated | -0.0225 | 0.0035 | -0.029393 | -0.01570 | 0 | -26.40 |
| COGLIM31 | rpart | indicated | -0.0265 | 0.0035 | -0.03339 | -0.01954 | 0 | -30.98 |
| HSELIM31 | forest | contraindicated | 0.0027 | 0.0048 | -0.006703 | 0.01210 | 0.5736 | 2.73 |
| HSELIM31 | rpart | contraindicated | 0.045 | 0.0045 | 0.03621 | 0.05384 | 0 | 45.5656 |
| HSELIM31 | forest | indicated | -0.0315 | 0.0035 | -0.038400 | -0.02464 | 0 | -31.90 |
| HSELIM31 | rpart | indicated | -0.0334 | 0.0035 | -0.04029 | -0.02653 | 0 | -33.81 |
| SCHLIM31 | forest | contraindicated | 0.0052 | 0.0042 | -0.003036 | 0.01336 | 0.2171 | 7.64 |
| SCHLIM31 | rpart | contraindicated | 0.0352 | 0.0039 | 0.02767 | 0.04279 | 0 | 52.16 |
| SCHLIM31 | forest | indicated | -0.0237 | 0.0031 | -0.029754 | -0.01761 | 0 | -35.06 |
| SCHLIM31 | rpart | indicated | -0.0242 | 0.0031 | -0.03023 | -0.01815 | 0 | -35.81 |
| SOCLIM31 | forest | contraindicated | 0.0145 | 0.0049 | 0.004956 | 0.02403 | 0.0029 | 16.13 |
| SOCLIM31 | rpart | contraindicated | 0.0535 | 0.0045 | 0.04465 | 0.06227 | 0 | 59.50 |
| SOCLIM31 | forest | indicated | -0.0203 | 0.0035 | -0.027201 | -0.01333 | 0 | -22.55 |
| SOCLIM31 | rpart | indicated | -0.0221 | 0.0035 | -0.02901 | -0.01514 | 0 | -24.58 |
| UNABLE31 | forest | contraindicated | 0.0054 | 0.0049 | -0.004228 | 0.01495 | 0.2731 | 5.16 |
| UNABLE31 | rpart | contraindicated | 0.0468 | 0.0046 | 0.03780 | 0.05584 | 0 | 45.04 |
| UNABLE31 | forest | indicated | -0.0347 | 0.0036 | -0.041750 | -0.02762 | 0 | -33.37 |
| UNABLE31 | rpart | indicated | -0.0374 | 0.0037 | -0.04461 | -0.03023 | 0 | -36.00 |
| WLKLIM31 | forest | contraindicated | 0.0185 | 0.0064 | 0.005976 | 0.03103 | 0.0038 | 8.95 |
| WLKLIM31 | rpart | contraindicated | 0.0796 | 0.006 | 0.06780 | 0.09131 | 0 | 38.46 |
| WLKLIM31 | forest | indicated | -0.0438 | 0.0047 | -0.052996 | -0.03457 | 0 | -21.16 |
| WLKLIM31 | rpart | indicated | -0.049 | 0.0048 | -0.05828 | -0.03961 | 0 | -23.66 |
| WRKLIM31 | forest | contraindicated | 0.0154 | 0.0057 | 0.004271 | 0.02654 | 0.0067 | 10.67 |
| WRKLIM31 | rpart | contraindicated | 0.0714 | 0.0053 | 0.06096 | 0.08175 | 0 | 49.41 |
| WRKLIM31 | forest | indicated | -0.0469 | 0.0042 | -0.055066 | -0.03863 | 0 | -32.44 |
| WRKLIM31 | rpart | indicated | -0.0519 | 0.0042 | -0.06024 | -0.04361 | 0 | -35.93 |
| ertot | forest | contraindicated | 0.0407 | 0.0136 | 0.01402 | 0.06736 | 0.0028 | 12.36 |
| ertot | rpart | contraindicated | 0.152 | 0.0127 | 0.1272 | 0.17677 | 0 | 46.16 |
| ertot | forest | indicated | -0.0793 | 0.0115 | -0.10177 | -0.05680 | 0 | -24.09 |
| ertot | rpart | indicated | -0.0958 | 0.0115 | -0.1183 | -0.07326 | 0 | -29.09 |
| ipdis | forest | contraindicated | 0.013 | 0.0082 | -0.003048 | 0.02908 | 0.1123 | 8.68 |
| ipdis | rpart | contraindicated | 0.0826 | 0.0078 | 0.06722 | 0.09799 | 0 | 55.09 |
| ipdis | forest | indicated | -0.048 | 0.0064 | -0.060588 | -0.03541 | 0 | -32.01 |
| ipdis | rpart | indicated | -0.0582 | 0.0066 | -0.07102 | -0.04531 | 0 | -38.79 |
| obtotv | forest | contraindicated | 1.068 | 0.2444 | 0.5886 | 1.5468 | 0 | 11.68 |
| obtotv | rpart | contraindicated | 3.5335 | 0.2331 | 3.077 | 3.9905 | 0 | 38.63 |
| obtotv | forest | indicated | -0.7 | 0.1859 | -1.0644 | -0.3356 | 0.0002 | -7.65 |
| obtotv | rpart | indicated | -0.8144 | 0.189 | -1.185 | -0.4439 | 0 | -8.90 |
| ztotexp | forest | contraindicated | 0.0794 | 0.0247 | 0.03105 | 0.1278 | 0.0013 | 1.00 |
| ztotexp | rpart | contraindicated | 0.3534 | 0.0226 | 0.3091 | 0.3977 | 0 | 4.45 |
| ztotexp | forest | indicated | -0.1867 | 0.0221 | -0.22994 | -0.1435 | 0 | -2.35 |
| ztotexp | rpart | indicated | -0.1727 | 0.0216 | -0.2150 | -0.1305 | 0 | -2.17 |
| Note: Estimates obtained from double machine learning estimates controlling for mean FDA approval year of prescribed drug, education, sex, age, race, year of MEPS survey, number of prescriptions and 477 medical conditions. Forest refers to random forest learners. Rpart refers to regression tree learners. ztotexpend: Total health care expenditure (log); IPDIS: Number of hospital inpatient discharges; ERTOT: Total number of emergency room visits; OBTOTV: Number of office-based provider visits. ANYLMT: = 1 if respondent has any activity limitations, =0 otherwise; ACTLIM31: = 1 if the respondent has activity limitations lasting 31 days or more, = 0 otherwise; UNABLE31: = 1 if the respondent has been unable to perform activities for 31 days or more; = 0 otherwise; WLKLIM31: = 1 if the respondent has limitations in walking lasting 31 days or more, = 0 otherwise; WRKLIM31: = 1 if the respondent has work limitations lasting 31 days or more, = 0 otherwise; SCHLIM31: = 1 if the respondent has school limitations lasting 31 days or more, = 0 otherwise; HSELIM31: = 1 if the respondent has home limitations lasting 31 days or more, = 0 otherwise; SOCLIM31: = 1 if the respondent has social activity limitations lasting 31 days or more, = 0 otherwise; COGLIM31: = 1 if the respondent has cognitive limitations lasting 31 days or more, = 0 otherwise. We used the broad definition of indicated prescription drug use. Data was obtained from the Medical Expenditure Panel Surveys 2016-2021, DrugCentral.org and the SNOMED classification. | | | | | | | | |

# 4. References

Bradford, W. David, John L. Turner, and Jonathan W. Williams. 2018. “Off-Label Use Of Pharmaceuticals: A Detection Controlled Estimation Approach.” *The Journal of Industrial Economics* 66 (4): 866–903. <https://doi.org/10.1111/joie.12189>.

Chernozhukov, Victor, Denis Chetverikov, Mert Demirer, Esther Duflo, Christian Hansen, Whitney Newey, and James Robins. 2018. “Double/Debiased Machine Learning for Treatment and Structural Parameters.” *The Econometrics Journal* 21 (1): C1–68. <https://doi.org/10.1111/ectj.12097>.

Probst, Philipp, Marvin Wright, and Anne-Laure Boulesteix. 2019. “Hyperparameters and Tuning Strategies for Random Forest.” *WIREs Data Mining and Knowledge Discovery* 9 (3): e1301. <https://doi.org/10.1002/widm.1301>.
